# A molecular phylogeny of Caraganeae (Leguminosae, Papilionoideae) reveals insights into new generic and infrageneric delimitations

**DOI:** 10.3897/phytokeys.70.9641

**Published:** 2016-10-04

**Authors:** Lei Duan, Xue Yang, Peiliang Liu, Gabriel Johnson, Jun Wen, Zhaoyang Chang

**Affiliations:** 1 Key Laboratory of Plant Resources Conservation and Sustainable Utilization, South China Botanical Garden, Chinese Academy of Sciences, Guangzhou, Guangdong 510650, P.R.China; 2 Agriculture School, Kunming University, Kunming, Yunnan 650204, P.R.China; 3 College of Life Sciences, Northwest A&F University, Yangling, Shaanxi 712100, China; 4 Department of Botany, National Museum of Natural History, MRC 166, Smithsonian Institution, Washington DC, 20013-7012, U.S.A.

**Keywords:** Caragana, Chesneya, Chesniella, chloroplast capture, generic delimitation, phylogeny

## Abstract

Based on sequence data of nuclear ITS and plastid *matK*, *trnL-F* and *psbA-trnH* markers, the phylogeny of the subtribes Caraganinae and Chesneyinae in tribe Caraganeae was inferred. The results support the monophyly of each of the subtribes. Within subtribes Caraganinae, *Calophaca* and *Halimodendron* are herein transferred into *Caragana* to ensure its generic monophyly. The subtribe Chesneyinae is composed of four well-supported genera: *Chesneya*, *Chesniella*, *Gueldenstaedtia* and *Tibetia*. Based on phylogenetic, morphological, distributional and habitat type evidence, the genus *Chesneya* was divided into three monophyletic sections: Chesneya sect. Chesneya, Chesneya sect. Pulvinatae and Chesneya sect. Spinosae. *Chesneya macrantha* is herein transferred into *Chesniella*. *Spongiocarpella* is polyphyletic and its generic rank is not maintained. The position of *Chesneya* was incongruent in the nuclear ITS and the plastid trees. A paternal chloroplast capture event via introgression is hypothesized for the origin of *Chesneya*, which is postulated to have involved the common ancestor of *Chesniella* (♂) and that of the *Gueldenstaedtia* – *Tibetia* (GUT) clade (♀) as the parents.

## Introduction

Caraganeae Ranjbar is a mid-sized tribe in Leguminosae, established by Ranjbar and Karamian (2003) based on five genera: *Calophaca* Fisch. ex DC., *Caragana* Fabr., *Chesneya* Lindl. ex Endl., *Gueldenstaedtia* Fisch. and *Halimodendron* Fisch. ex DC., numbers of genera may be altered when treated by different workers (see below). Caraganeae ranges from eastern Europe, central and western Asia to Mongolia, China and the Himalayas, extending northward to Siberia (Lock 2005; Ranjbar et al. 2014). This tribe is diagnosed by the asymmetrical axillary peduncles or pedicels attached to the slightly gibbous calyx and dehiscent pods (except for *Halimodendron*; Polhill 1981; Ranjbar and Karamian 2003; Ranjbar et al. 2014).

A few recent studies referred to the concept of Caraganeae. Molecular work of Ranjbar et al. (2014) classified Caraganeae into two subtribes: Caraganinae and Chesneyinae Ranjbar, F. Hajmoradi & Waycott. Duan et al. (2015) recognized this tribe based on the genera *Calophaca*, *Caragana* and *Halimodendron*. However, the former was inferred from a limited sampling scheme and few DNA markers, while the latter was subject to the undersampled for Chesneyinae. Hence, the monophyly of this tribe and the division of subtribes need to be further evaluated.

Within the subtribe Caraganinae, the genus *Caragana* has attracted much attention (Komarov 1908; Moore 1968; Gorbunova 1984; Zhao 1993, 2009; Zhou 1996; Zhang 1997; Sanchir 1999; Sanczir 2000; Hou et al. 2008; Zhang et al. 2009). The infrageneric classifications of *Caragana* mainly focused on several morphological characters: leaves paripinnate vs. digitate, with four vs. more leaflets, and petioles and rachises caducous vs. persistent. Recent phylogenetic analyses resolved that *Caragana* was paraphyletic, with *Halimodendron* and *Calophaca* embedded in it (Zhang et al. 2009, 2015a; Zhang and Fritsch 2010; Duan et al. 2015). Thus, proposal of a new generic delimitation for *Caragana* may be possible based on more comprehensive phylogenetic evidence.

The genera *Chesneya* and *Gueldenstaedtia* formed a well-supported clade (Sanderson and Wojciechowski 1996), and were treated as the subtribe Chesneyinae (Ranjbar et al. 2014). Within this subtribe, the generic delimitations were controversial, especially concerning the status of *Chesniella* Boriss. (Borissova 1964), *Spongiocarpella* Yakovl. et Ulzij. (Yakovlev and Sviazeva 1987), and *Tibetia* (Ali) H. P. Tsui (Tsui 1979). The former two genera were separated from *Chesneya*, while *Tibetia* was a segregate of *Gueldenstaedtia* and has been revised in several studies (Cui 1998; Zhu 2004; Zhu 2005a, 2005b; Bao and Brach 2010). Zhang et al. (2015b) supported the monophyly of *Chesneya* and proposed a classification system, but some sections were only weakly supported. Hence, the phylogeny of Chesneyinae and its associated genera needs to be further explored.

We herein employ sequence data from nrDNA ITS and plastid *matK*, *trnL-F* and *psbA-trnH* to a) test the monophyly of Caraganeae and its subtribes; b) estimate the phylogeny of genera in Caraganeae; and c) discuss the taxonomic implications of this phylogeny on the generic and the infrageneric classification of the tribe.

## Materials and methods

### Taxon sampling

Our sampling was designed largely following the generic demarcations in *Flora Reipublicae Popularis Sinicae* (Liou 1993; Li 1993; Cui 1998). We included 101 accessions, covering 97 species, containing 39 species of Caraganinae (represented by *Calophaca*, *Halimodendron* and all 5 sections of *Caragana* according to Zhang 1997) and 40 accessions (36 species) of Chesneyinae (including *Chesneya*, *Chesniella*, *Gueldenstaedtia* and *Tibetia*, tentatively treating *Spongiocarpella* in *Chesneya*, which were more comprehensively sampled than previous studies [Ranjbar et al. 2014; Duan et al. 2015; Zhang et al. 2015b]). 82 new sequences were generated in this work.

To better resolve the relationships of subtribes Caraganinae and Chesneyinae, 11 Galegeae species (8 genera) and 5 Hedysareae species (4 genera) were also sampled. *Cicer microphyllum* Royle ex Bentham, *Dalbergia hupeana* Hance, *Lathyrus latifolius* L., *Robinia pseudoacacia* L., *Trifolium repens* L. and *Wisteria sinensis* (Sims) Sweet were selected as outgroups based on previous studies (Wojciechowski et al. 2000, 2004; Wojciechowski 2003). Sequences of 40 accessions (representing 40 species) were downloaded from GenBank (see Suppl. material 1 for details). Most accessions we sampled were collected from the field or herbarium specimens. *Onobrychis arenaria* DC. was obtained from seedlings germinated from seeds provided by the Royal Botanic Gardens, Kew.

### DNA extraction, amplification and sequencing

Total genomic DNAs were extracted from silica-gel dried leaves or herbarium material using the Plant DNA Extraction Kit - AGP965/960 (AutoGen, Holliston, MA, USA) or the DNeasy Plant Mini Kit (Qiagen, Valencia, USA). Polymerase chain reactions (PCR) were prepared in 25µL containing 1.5 mM MgCl_2_, 0.2 mM of each dNTP, 0.4 mM of each primer, 1 U of *Taq* polymerase (Bioline, Aberdeen, Scotland, UK), and using 10–50 ng (2.5 µL) template DNAs, following Wen et al. (2007). The PCRs for ITS (primer pair: ITS4 and ITS5a) and *psbA-trnH* (primer pair: psbA and trnH) were performed according to Stanford et al. (2000) and Hamilton (1999), respectively. The PCR primer pair for *trnL-F* was “c” and “f” as in Zhu et al. (2013) and Taberlet et al. (1991), and the thermal cycling program followed Soejima and Wen (2006). The barcoding region of the *matK* marker was amplified and sequenced with the primer pair Kim-3F/Kim-1R (CBOL Plant Working Group 2009; China Plant BOL Group 2011), and the amplification conditions were: 95°C (5min) for DNA pre-denaturation; 94°C (40s), 48°C (40s) and 72°C (100s) for 35 cycles; 72°C (10min) for final extension. PCR products were cleaned using ExoSAP-IT (cat. # 78201, USB Corporation, Cleveland, OH, USA) following the manufacturer’s instruction. Purified products were sequenced from both directions with BigDye 3.1 reagents on an ABI 3730 automated sequencer (Applied Biosystems, Foster City, CA, USA).

### Phylogenetic analysis

Sequences were assembled with Geneious 7.1 (http://www.geneious.com/), and aligned using MUSCLE 3.8.31 (Edgar 2004), followed by manual adjustments in Geneious 7.1. Because the chloroplast markers putatively evolve as a single molecule, sequences of the three plastid markers (*matK*, *trnL-F* and *psbA-trnH*) were directly concatenated. Topological discordance was investigated by comparing the ITS and the concatenated plastid trees (as in García et al. 2014). To further determine the compatibility between these two datasets, an incongruence length difference (ILD) test and an approximately unbiased (AU) test were conducted with PAUP* (Swofford 2003) and CONSEL (Shimodaira and Hasegawa 2001; using site-wise likelihood values estimated by RA×ML; Stamatakis et al. 2008) programs, respectively. The tests retrieved the *p* values of 0.01 and 0.0001, respectively, suggesting that the incongruence between these two datasets was significant. The ITS and the concatenated plastid sequences were thus analyzed separately.

Phylogenetic analyses were carried out using Bayesian inference (BI; Rannala and Yang 1996; Mau et al. 1999) with MrBayes 3.2.5 (Ronquist and Huelsenbeck 2003; Ronquist et al. 2012). Nucleotide substitution model parameters were determined prior to BI using the corrected Akaike information criterion (AIC) in jModeltest 2.1.7. (Posada 2008; Darriba et al. 2012). For the ITS dataset, boundaries of the 5.8S region to the ITS1 and the ITS2 regions were determined by comparison with the published 5.8S sequence of *Vicia faba* L. (Nazar and Wildeman 1981; Yokota et al. 1989), and the sequence substitution models for the ITS1, 5.8S and ITS2 regions were determined separately. Similarly, the models for each of the three plastid markers were estimated for the best-fit models, which were used in the BI analysis for concatenated plastid sequences in a partitioned scheme.

In the BI, the Markov chain Monte Carlo (MCMC) search was run by two replicates for 10,000,000 generations, sampling one tree every 1,000 generations. After the first 2,500,000 generations (2,500 trees) were discarded as burn-in, a 50% majority-rule consensus tree and posterior probabilities were obtained among the remaining trees. Results were checked using the program Tracer 1.5 (Rambaut and Drummond 2007) to ensure that plots of the two runs were converging and the value of the effective sample size for each replicate was above 200. Maximum likelihood (ML) analyses were conducted using RAxML-MPI v8.2 (Stamatakis 2014) with dataset partition scheme the same as in the BI and the following settings: rapid bootstrap analysis with 1,000 replicates and search for best-scoring ML tree in one program run, starting with a random seed, selecting the GTR model. Bootstrap values (LBS), as well as posterior probabilities (PP) were labeled on the corresponding branches of the Bayesian trees.

## Results

**Figure 1.**
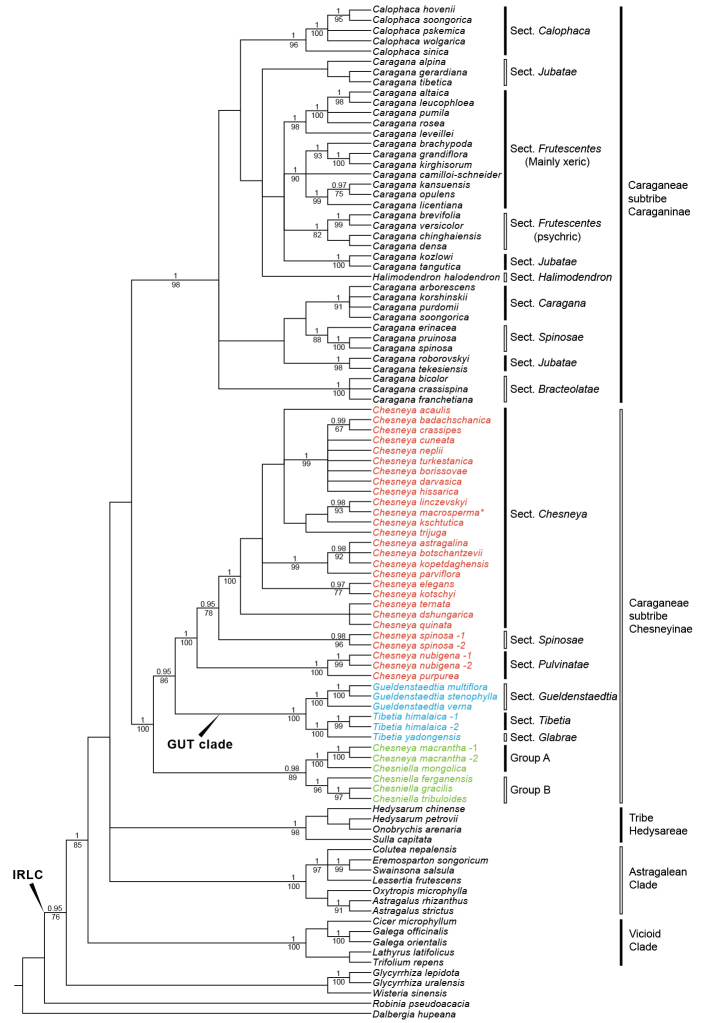
Bayesian tree of the nrDNA ITS data, showing relationships of genera in subtribes Caraganinae, Chesneyinae and their close relatives. The labeled sections of *Gueldenstaedtia* and *Tibetia* followed Tsui (1979) and Zhu (2005a), respectively. Bayesian posterior probabilities (PP ≥ 0.95) and maximum likelihood bootstrap (LBS ≥ 70%) are given above and below branches, respectively. The asterisk indicates the name of *Chesneya macrosperma* has not been published, its voucher was storied in LE (details see Zhang et al. 2015b).

**Figure 2.**
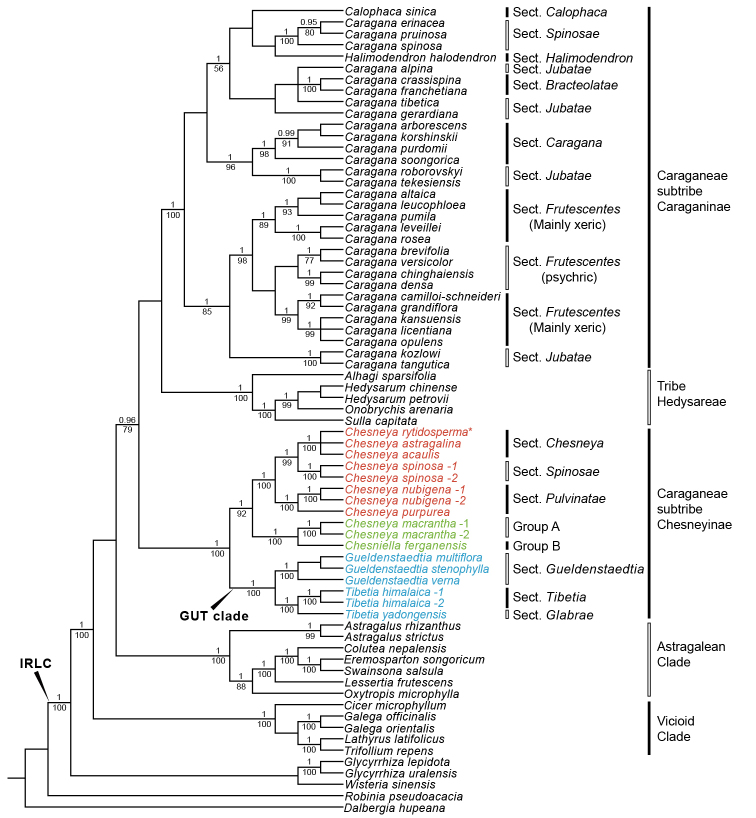
Bayesian tree of the concatenated plastid data of *matK*, *trnL-F* and *psbA-trnH* sequences, showing genera in subtribes Caraganinae, Chesneyinae and their close relatives. The labeled sections of *Gueldenstaedtia* and *Tibetia* followed Tsui (1979) and Zhu (2005a), respectively. Bayesian posterior probabilities (PP ≥ 0.95) and maximum likelihood bootstrap (LBS ≥ 70%) are given above and below branches, respectively. The asterisk indicates the type species of *Chesneya*.

Sequence characteristics are shown in Table 1. Our ML results are basically congruent in topology with the corresponding BI trees, the support values of the former were thus labeled on the corresponding branches of the latter (see legend of Figs 1, 2). Thanks to some extra sequences from GenBank (see Suppl. material 1), especially those of *Chesneya* and *Chesniella*, the ITS tree (Fig. 1) was more comprehensively sampled than the plastid tree (Fig. 2), which was of help to increase the general support of the former.

**Table 1.** Sequence characteristics with gaps as missing data: alignment length, the number of the constant, variable and potential parsimony-informative (Pi) sites, and the best-fit nucleotide substitution model determined by AIC.

Dataset	Length	Constant	Variable	Pi	Model
ITS1	266	81	185	148	GTR+I+G
5.8S	164	143	21	14	TrNef+I+G
ITS2	279	113	166	131	GTR+G
*matK*	807	485	322	189	GTR+G
*trnL-F*	1412	921	491	279	TVM+I+G
*psbA-trnH*	793	472	321	175	TIM1+G

### Nuclear data

In the ITS tree (Fig. 1), the Astragalean clade (PP = 1, LBS = 100%; including *Astragalus* L., *Colutea* L., *Eremosparton* Fisch. & C.A.Mey., *Lessertia* R.Br. ex W.T.Aiton, *Oxytropis* DC., and *Swainsona* Salisb.), the Vicioid clade (PP = 1, LBS = 100%; represented by *Trifolium*, *Lathyrus*, *Cicer* and *Galega* L.), tribe Hedysareae (PP = 1, LBS = 98%), subtribes Caraganinae (PP = 1, LBS = 98%) and Chesneyinae (PP = 1, LBS = 100%) were each strongly supported.

Subtribe Caraganinae contained three genera, within which *Calophaca* was monophyletic (PP = 1, LBS = 96%), but *Calophaca* and *Halimodendron* were embedded within the paraphyletic *Caragana*. Within subtribe Chesneyinae, *Gueldenstaedtia* (PP = 1, LBS = 100%) and *Tibetia* (PP = 1, LBS = 100%) were each monophyletic and together they formed a clade (the GUT clade, shown in blue; PP = 1, LBS = 100%). Two accessions of former *Chesneya macrantha* Cheng f. ex H.C.Fu constituted a robustly supported branch nested in a monophyletic *Chesniella* (displayed in green; PP = 0.98, LBS = 89%), while other accessions of *Chesneya* formed another clade (*Chesneya* *s.s.*; shown in red; PP = 1, LBS = 100%; Fig. 1), which contained three well-supported sections (details see Discussion; PP = 1 & LBS = 100%, PP = 0.98 & LBS = 96% and PP = 1 & LBS = 100%, respectively).

### Plastid data

Similar to the ITS results, the plastid tree (Fig. 2) also showed the monophyly of both subtribes Caraganinae (PP = 1, LBS = 100%) and Chesneyinae (PP = 1, LBS = 100%). *Calophaca* and *Halimodendron* were nested in *Caragana* in different places from the ITS tree, but such placement was weakly supported. *Caragana* also showed its paraphyly, with Chesneya sect. Bracteolatae (Kom.) M.L.Zhang (PP = 1, LBS = 100%), Chesneya sect. Caragana Kom. (PP = 1, LBS = 98%), Chesneya sect. Frutescentes (Kom.) Sanchir (PP = 1, LBS = 98%) and Chesneya sect. Spinosae (Kom.) Y.Z.Zhao (PP = 1, LBS = 100%) each strongly supported. Unlike in the ITS tree, *Chesneya* *s.s.* and *Chesniella* were sisters in the plastid tree (PP = 1, LBS = 92%; Fig. 2). As in the ITS tree, the GUT clade (PP = 1, LBS = 100%) contained *Gueldenstaedtia* (PP = 1, LBS = 100%) and *Tibetia* (PP = 1, LBS = 100%), with each genus being monophyletic.

## Discussion

Caraganeae comprises ca. 100 species distributed in temperate Asia, extending to eastern Europe (Ranjbar and Karamian 2003; Lock 2005). The two subtribes (Caraganinae and Chesneyinae) recognized by Ranjbar et al. (2014) are each well-supported in our analyses. However, our results did not recover a monophyletic Caraganeae (Figs 1, 2). Similarly, the previously expanded delimitation of Hedysareae *sensu* Lock (2005; also see Cardoso et al. 2013), which included the genera of subtribe Caraganinae and tribe Hedysareae *sensu* Amirahmadi et al. (2014), is not confirmed herein (Figs 1, 2).

Subtribe Caraganinae is composed of *Calophaca*, *Caragana* and *Halimodendron* (Ranjbar et al. 2014). Morphologically, this subtribe differs from Chesneyinae by several characters, including habit (shrubs vs. perennial herbs or subshrubs), leaf type (paripinnate [except for *Calophaca*] vs. imparipinnate) and nerve type on wing petals (pinnate vs. palmate except for *Chesneya*; Lock 2005; Ranjbar et al. 2014; Duan et al. 2015). Caraganinae is also distinct from Hedysareae (as delimited in Amirahmadi et al. 2014 and Duan et al. 2015) based on the following morphological characters: shrubs, rarely small trees; paripinnate, rarely imparipinnate leaves (*Calophaca*); solitary flowers, or a few flowers in fascicles, rarely forming a raceme; pods cylindric, rarely compressed, glabrous or hairy, with dehiscent and twisted valves (except for *Halimodendron*; Polhill 1981; Liu et al. 2010b). Caraganinae is also related to the Astragalean clade; yet due to the morphological diversity of the latter, there are few diagnosable features to differentiate the Astragalean clade from Caraganinae, except for the twisted valves of Caraganinae (*Calophaca* and *Caragana*).

### An expanded generic concept of *Caragana*

Within Caraganinae, *Halimodendron* contains only *Halimodendron halodendron* (Pall.) Druce with its distribution roughly overlapping with that of *Calophaca* (Lock 2005). This species is morphologically unique in Caraganinae with its inflated pods (Gorshkova 1945; Liu et al. 2010b). Consistent with previous studies (Zhang et al. 2009; Zhang and Fritsch 2010), our results also showed that *Halimodendron* is nested within *Caragana*. The phylogenetic evidence hence supports treating *Halimodendron* as a section within *Caragana*, i.e., Caragana sect. Halimodenron (Fisch. ex DC.) L.Duan, J.Wen & Zhao Y.Chang. We also resurrect the name *Caragana halodendron* (Pallas) Dumont de Courset based on *Halimodendron halodendron* (Figs 1, 2; see Taxonomic Treatment).

*Calophaca* morphologically resembles *Caragana*, and it is only distinguished from the latter by its imparipinnate leaves, rachises without thorns, and relatively denser racemes (Borissova 1945; Liu et al. 2010b). *Calophaca* contains 5–8 species mainly distributed in mountainous areas of central Asia, with one species extending to eastern Europe, and one endemic to northern China (Borissova 1945; Tutin et al. 1968; Yakovlev et al. 1996; Lock 2005; Liu et al. 2010b; Zhang et al. 2015a). The embedded position of *Calophaca* within *Caragana* argues that its classification needs to be placed in the broader phylogenetic framework of *Caragana*, which is supported by our results (Figs 1, 2) and several previous studies (e.g., Zhang et al. 2009, 2010, 2015a, b; Duan et al. 2015). We thus merge *Calophaca* into *Caragana* and recognize it at the sectional level as Caragana sect. Calophaca (Fisch. ex DC.) L.Duan, J.Wen & Zhao Y.Chang (see Taxonomic Treatment). The species-level nomenclatural changes will be made in a follow-up paper.

The taxonomy of *Caragana* has been investigated by various authors (Komarov 1908; Poyarkova 1945; Moore 1968; Sanczir 1979, 2000; Gorbunova 1984; Zhao 1993; Zhou 1996; Zhang 1997; Sanchir 1999; Chang 2008). However, *Caragana* *s.s.* as previously circumscribed is clearly paraphylytic (Zhang et al. 2009; Duan et al. 2015). We herein propose the delimitation of *Caragana* *s.l.* to ensure the generic monophyly (see Taxonomic Treatment). *Caragana* as defined now contains taxa of *Calophaca*, former *Caragana* *s.s.* and *Halimodendron* (Figs 1, 2), which is classified into seven sections: Caragana sect. Bracteolatae M.L.Zhang, Caragana sect. Calophaca, Caragana sect. Caragana, Caragana sect. Frutescentes (Kom.) Sancz., Caragana sect. Halimodenron, Caragana sect. Jabatae (Kom.) Y.Z.Zhao and Caragana sect. Spinosae (Kom.) Y.Z.Zhao. Although *Caragana* *s.l.* is morphologically diverse, this genus can be diagnosed by its shrubby habit, saccate, oblique calyx bases, pinnate nerves on the wing petals and twisted, dehiscent pods (except for *Caragana holodendron*). The expanded concept of *Caragana* is also supported by cytological evidence (Moore 1968; Chang 1993; Li 1993; Zhou et al. 2002; Chang 2008): most xeric and psychric taxa of *Caragana* *s.l.* have the same basic chromosome number (*x* = 8).

At the sectional level, our ITS tree (Fig. 1) indicated a strongly supported Caragana sect. Calophaca. On the other hand, former *Caragana* *s.s.* was divided into five sections mainly based on the combinations of leaf (pinnate or digitate) and petiole/rachis (persistent or caducous) characters (Zhang 1997). Three main sections, Caragana sect. Bracteolatae, Caragana sect. Caragana and Caragana sect. Frutescentes, evolved likely accompanying the rapid uplifts of the Qinghai-Tibet Plateau (QTP) at around 8 Ma (Zhang et al. 2009). These three sections also largely correspond to psychrophytic, mesophytic and xerophytic habitats, respectively (Zhang and Fritsch 2010). Our analyses supported the monophyly of the three sections, with Caragana sect. Frutescentes only being monophyletic in the plastid tree (also see Zhang et al. 2009; Duan et al. 2015; and see below for an exceptional case in Caragana sect. Frutescentes). Our ITS results failed to resolve a monophyletic Caragana sect. Frutescentes (Fig. 1), but this may be due to insufficient informative sites in the ITS data. Furthermore, we only sampled one series for Caragana sect. Spinosae (Caragana ser. Spinosae Kom.), thus cannot assess its monophyly (Figs 1, 2). Caragana sect. Jabatae was suggested to have experienced a rapid radiation at 3.4–1.8 Ma (Zhang and Fritsch 2010), which may partly explain its poorly resolved relationships in our trees (Figs 1, 2; also see Zhang et al. 2009; Duan et al. 2015).

At the infra-sectional level, Caragana ser. Bracteolatae Kom. and Caragana ser. Spinosae are well-supported by our results (not labeled in the trees). Our results are therefore not completely congruent with Zhang et al. (2009), possibly due to differences in taxon sampling. Interestingly, a strongly supported psychric group is found within the mainly xeric section Caragana sect. Frutescentes (Zhao 2009). This group is represented by *Caragana brevifolia* Kom., *Caragana chinghaiensis* Y.X.Liou, *Caragana densa* Kom. and *Caragana versicolor* Benth. (in Fig. 1; but weakly supported in the plastid tree). Most species of Caragana sect. Frutescentes range from eastern Europe to northern China, Mongolia and Siberia, however, this above-mentioned psychric group is distributed in the southern edge of northern China, extending to Tibet and its neighboring regions. It may represent a vicariant transitional group of Caragana sect. Bracteolatae, Caragana sect. Jubatae pro parte, Caragana sect. Spinosae pro parte (psychrophytic habitat) and Caragana sect. Frutescentes. Other cases of vicariant distributions have been noted in *Caragana*, and vicariance was considered as an important biogeographic pattern for this genus. For example, three closely related species in Caragana sect. Caragana, *Caragana microphylla* Lam., *Caragana intermedia* Kuang & H.C.Fu and *Caragana korshinskii* Kom., show non-overlapping to only slightly overlapping distributions in northeast to northwest China (Shue and Hao 1989; Zhang and Wang 1993; Zhang 1998; Chang 2008).

### Phylogeny of Chesneyinae

The subtribe Chesneyinae, as established by Ranjbar et al. (2014), was supported to be monophyletic in our trees (Figs 1, 2). Three main clades can be recognized within this subtribe: the GUT clade, *Chesneya* *s.s.* and *Chesniella* (Figs 1, 2).

**Figure 3.**
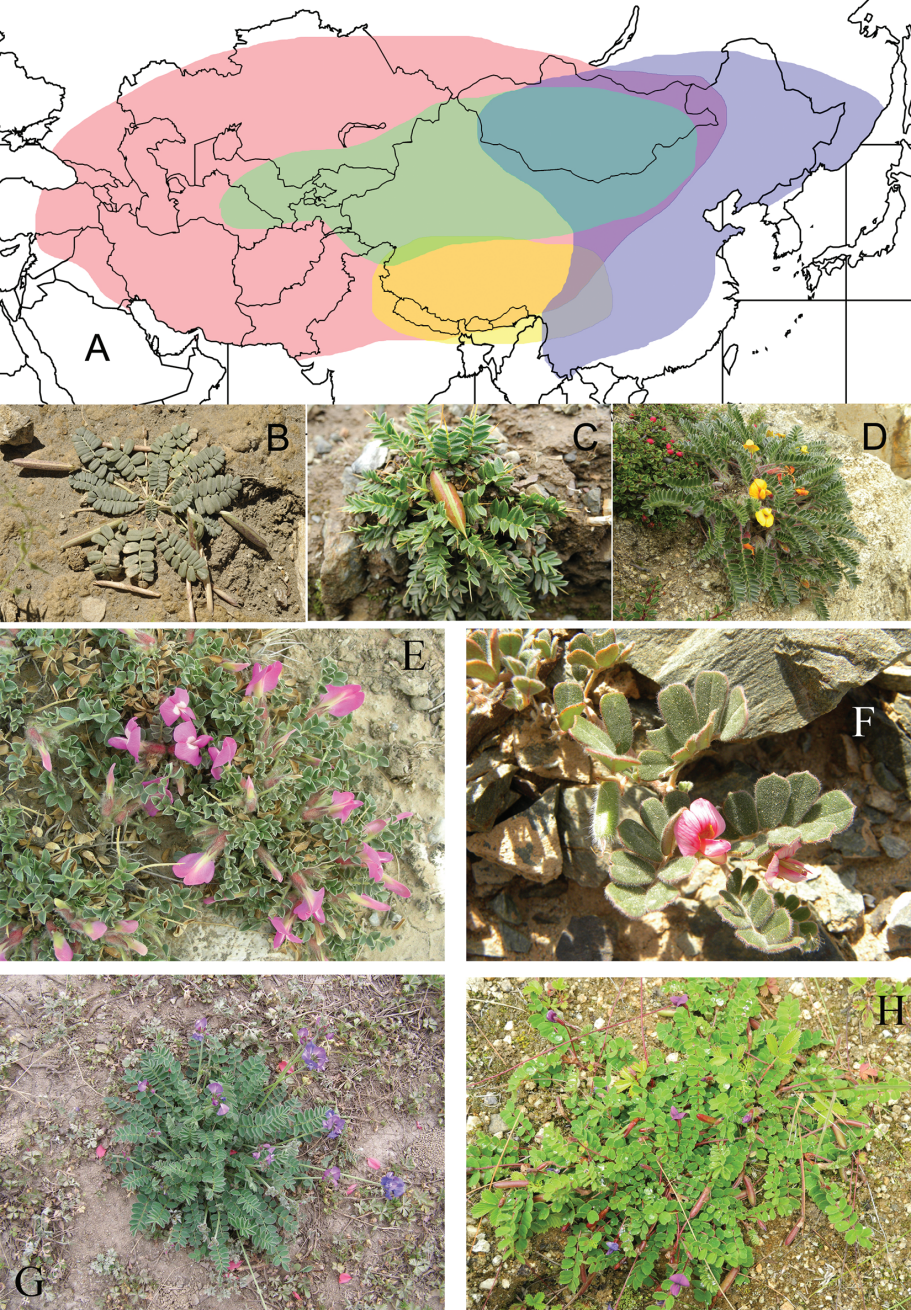
Distribution (**A**) and representative plants (**B–H**) of genera in Chesneyinae. **A** red – *Chesneya*, green – *Chesniella*, blue – *Gueldenstaedtia* and yellow – *Tibetia* **B** *Chesneya acaulis* **C** *Chesneya spinosa* **D** *Chesneya nubigena* **E** *Chesniella macrantha* **F** *Chesniella ferganensis* **G** *Gueldenstaedtia verna* **H** *Tibetia yadongensis*.

This subtribe contains ca. 50 species and differs from the Astragalean clade by twisted valves (e.g., in *Chesneya*), but a few species of *Astragalus* also have twisted legumes. Taxa of Chesneyinae are distinguished from Hedysareae by their dehiscent pods (Borissova 1945; Yakovlev et al. 1996; Liu et al. 2010a). The genera of Chesneyinae are distributed in central and eastern Asia, Tibet, Mongolia and Siberia, extending to eastern Turkey and Armenia (Fig. 3A; Borissova 1945; Davis 1970; Rechinger 1984; Lock and Schrire 2005; Liu et al. 2010a), which are largely adapted to xerophytic (*Chesneya* and *Chesniella*), mesophytic (*Gueldenstaedtia*) and psychrophytic (*Tibetia*) habitats, respectively, although some species of *Chesneya* (see discussion below) and a few of *Gueldenstaedtia* are psychric taxa. The uplift of the QTP and aridification of the former Tethys region might have driven the origination and divergence of genera in the subtribe Chesneyinae (Wen et al. 2014; Meng et al. 2015; Zhang et al. 2015b).

### Topological discordance between ITS and plastid trees in subtribe Chesneyinae

The ITS and plastid topologies are incongruent within Chesneyinae. *Chesneya* *s.s.* formed a clade with the GUT clade in the ITS tree (Fig. 1), whereas it was sister to *Chesniella* in the plastid tree (Fig. 2). Both relationships were well-supported. Various mechanisms have been proposed to explain discordant topologies between gene trees, such as allopolyploidy, hybridization, horizontal gene transfer, incomplete lineage sorting (ILS), different rate of molecular evolution, and chloroplast capture (Degtjareva et al. 2012; García et al. 2014; Yi et al. 2015).

Allopolyploidy can be ruled out for two reasons. First, taxa within Chesneyinae are diploid (Nie et al. 2002; Yang 2002; Sepet et al. 2014), with no evidence of polyploidy in this subtribe and its allied tribes. Second, deep lineages of Chesneyinae basically display a consistent chromosome number (*x* = 8; Nie et al. 2002; Sepet et al. 2014), with the only exception of *Gueldenstaedtia* (*x* = 7; Yang 2002), which has relatively recently diverged (ca. 15.23 Ma; Zhang et al. 2015b).

ILS and chloroplast capture seem more likely mechanisms for the present case (Tsitrone et al. 2003; Deng et al. 2015; Sun et al. 2015). A time-calibrated phylogeny may facilitate the exploration of the likely mechanism. Incomplete lineage sorting, which rarely occurs in deep lineage (Sun et al. 2015), prevails with bifurcation patterns of the shallow lineages of gene trees (especially at the specific level; Xu et al. 2012), and usually takes place in groups with relatively recent diversification times (García et al. 2014). Zhang et al. (2015b) estimated that the main clades of subtribe Chesneyinae split at ca. 28 Ma, which is beyond the time frame supporting ILS of ancestral polymorphisms (as suggested by Xu et al. 2012). On the other hand, biogeographic patterns can also be taken into consideration (Goodman et al. 1999). Given peripatry and parapatry may have been involved in the evolution of Chesneyinae, if ILS occurred, the main clades would hardly be resolved with well-supported dichotomy as presented herein. Hence, although ILS could not be completely excluded in this case, we regarded chloroplast capture as the most likely cause for the discordant position of *Chesneya* *s.s.*

Compared to the biparental inheritance of the nuclear genome, plastid DNA of angiosperms is usually uniparentally transmitted, especially maternally (Corriveau and Coleman 1988; McCauley et al. 2007; Wicke et al. 2011). Nevertheless, the plastid DNA of the inverted repeat lacking clade (IRLC; see Figs 1, 2; also as in Lavin et al. 1990; Wojciechowski et al. 2000) in Leguminosae was reported to be inherited paternally or biparentally (Zhang et al. 2003), confirmed by cytoplasmic and phylogenetic studies focusing on *Medicago* L. (paternal transmission; Schumann and Hancock 1989; Masoud et al. 1990; Havananda et al. 2010) and *Wisteria* Nutt. (Hu et al. 2005; Trusty et al. 2007). As *Chesneya* *s.s.* belongs to IRLC, a paternal inheritance scenario might be the case for the plastid DNA of *Chesneya* *s.s.*

We herein hypothesize a chloroplast capture event in the origin of *Chesneya* *s.s.* as follows. The common ancestor of *Chesniella* served as the putative paternal parent of *Chesneya* *s.s.* (sister to *Chesneya* *s.s.* in the plastid tree; Fig. 2). The maternal parent most likely was the common ancestor of the GUT clade. Their hybrids, with plastid from the paternal parent, may have continuously backcrossed with the maternal parent, and led to *Chesneya* *s.s.* inheriting most of the nuclear genome maternally (Fig. 1). Such a chloroplast capture event via introgression likely took place in the Miocene, because the divergence of *Chesneya* *s.s.* was dated to be 16.56 Ma and that of *Chesniella* was estimated as 19.81 Ma (Zhang et al. 2015b).

Analyses of Zhang et al. (2015b) revealed that the divergence of *Chesneya* and *Chesniella* most likely occurred around the QTP. Our analysis further indicated the psychric group of *Chesneya* diverged first in this genus (Chesneya sect. Pulvinatae, see Discussion below). It is probable that the common ancestor of *Chesniella* adapted to psychrophytic habitats. However, the extant *Chesniella* is rarely distributed on the QTP. As for the GUT clade, *Gueldenstaedtia* possesses a unique chromosome number (x = 7; Yang 2002) within the subtribe. Most species of *Gueldenstaedtia* are adapted to mesophytic habitats of temperate northern and eastern Asia (Fig. 3A), in contrast to the rest of Chesneyinae, which are psychric or xeric taxa. Such a correlation among the variation of chromosome numbers and adaptation to different habitats has also been recorded in other taxa, such as *Hedysarum* (Tang 2005; Duan et al. 2015), *Passiflora* (Hansen et al. 2006) and Amaryllidaceae (García et al. 2014). But the mechanisms of these types of adaptation need to be further explored with robust phylogenetic, ecological and biogeographic analyses in our future efforts.

### Phylogeny and treatment of *Chesneya*, *Chesniella* and *Spongiocarpella*

*Chesneya* is the type genus of Chesneyinae, with ca. 35 species (see Fig. 3B–D). This genus has its distribution from the Himalayan region to northwestern China and Mongolia, through central and western Asia, westward to Turkey and Armenia (Fig. 3A; Borissova 1945; Davis 1970; Yakovlev et al. 1996; Lock and Schrire 2005; Fig. 3A). Our results suggest that the formerly circumscribed *Chesneya*, which contains two well-supported but separated parts: the core *Chesneya* *s.s.* and the outlier *Chesneya macrantha* (Fig. 3E) (as in Li 1993 & Zhu and Larsen 2010), is not monophyletic (Figs 1, 2). *Chesneya spinosa* P.C.Li (Fig. 3C) of *Chesneya* *s.s.* is morphologically similar to *Chesneya macrantha* (Li 1981). However, *Chesneya spinosa* is distributed in southern Tibet, while *Chesneya macrantha* is restricted to the dry lands of Mongolia and northwestern China (Li and Ni 1985; Fu 1989). They occupy psychrophytic and xerophytic habitats, respectively, and are clearly not sister to each other (Figs 1, 2).

*Chesneya macrantha* is nested within a monophyletic *Chesniella* according to our ITS tree (Fig. 1), and in the plastid tree, it is sister to the type of *Chesniella*: *Chesniella ferganensis* (Korsh.) Boriss. (Borissova 1964; see Fig. 2, 3F). *Chesneya macrantha* shows some distinct morphologies from the other species in *Chesniella*, including its pulvinate habit and persistent leaf rachis (Li 1993), but this species generally share distribution areas, xerophytic habitats, and some synapomorphies, such as membranous stipules, hairy standard and ovate leaflets with cuneate apices, with *Chesniella* (Li and Ni 1985; Fu 1989; Zhu and Larsen 2010). Therefore, the transfer of *Chesneya macrantha* to *Chesniella* is supported by morphological, geographic and phylogenetic evidence (see Taxonomic Treatment). On the other hand, *Chesneya* was thus re-delimited based on the monophyletic *Chesneya* *s.s.*

After its establishment by Lindley (1839), *Chesneya* was divided into Chesneya sect. Macrocarpon Boriss. and Chesneya sect. Microcarpon Boriss. mainly based on pod morphology (Borissova 1945). The latter was segregated as the genus *Chesniella* by Borissova (1964), and this treatment was followed by Li (1993) and Zhu and Larsen (2010). Zhang et al. (2015b) informally classified *Chesneya* into five sections without detailed taxonomic treatment. Not all their sections were monophylytic, and the diagnostic characters and distributions of several sections were overlapping to some extent.

The presently demarcated *Chesneya* was assigned into three strongly supported sections herein (as in the key of *Chesneya* proposed by Li 1993; details see Figs 1, 2 and Taxonomic Treatment). Chesneya sect. Macrocarpon possesses non-pulvinate habit, reduced stems, truncate or emarginate leaflet apices and caducous petiole and rachis (Borissova 1945). This section is composed of most species of *Chesneya*, including the type species: *Chesneya rytidosperma* Jaub. et Spach (see Fig. 2; Borissova 1945; Davis 1970; Rechinger 1984). Chesneya sect. Macrocarpon was thus treated as Chesneya sect. Chesneya (Fig. 3B). Unlike this section, petioles and rachises of Chesneya sect. Pulvinatae M.L.Zhang (Zhang et al., 2015b; see Fig. 3D) are persistent and pubescent. However, most species in Chesneya sect. Pulvinatae have blackened and curved petioles and rachises, while those of one of its species, *Chesneya spinosa*, are hardened and spiny. Besides, *Chesneya spinosa* formed a clade separated from Chesneya sect. Pulvinatae. Hence, it is appropriate to segregate this species to form a new monotypic section: Chesneya sect. Spinosae L.Duan, J.Wen & Zhao Y.Chang (see see Fig. 3C and Taxonomic treatment).

The infra-sectional relationships within Chesneya sect. Chesneya are basically unresolved in our ITS trees (Fig. 1), and this section is undersampled in the plastid trees (Fig. 2). As for Chesneya sect. Pulvinatae, two accessions of *Chesneya nubigena* (D.Don) Ali formed a clade, being sister to *Chesneya purpurea* P.C.Li (Figs 1, 2). Based on such well-supported tree topologies and several morphological differences, such as smaller leaflets and purple corollae, the specific status of *Chesneya purpurea* was retained herein (as in Li 1981, 1993).

The xeric Chesneya sect. Chesneya grows on dry slopes or desert margins of northwestern China, Mongolia and central Asia (see Fig. 3B; Borissova 1945; Rechinger 1984; Lock and Simpson 1991; Yakovlev et al. 1996; Zhu and Larsen 2010). This section is morphologically similar to *Chesniella* (Fig. 3F) and their distributions are more or less overlapping (Borissova 1945; Li, 1993), whereas they are not phylogenetically close to each other (Figs 1, 2). Such a phenomenon may be due to convergent evolution (Degtjareva et al. 2012). Chesneya sect. Spinosae (Fig. 3C) and Chesneya sect. Pulvinatae (Fig. 3D) are restricted to Tibet and adjacent regions, adapting to high-altitude psychrophytic habitats (Ali 1977; Zhu and Larsen 2010). The evolutionary history of *Chesneya* appears complex, whereas the elevation of the QTP and the subsequent aridifications may have played an important role (Meng et al. 2015; Zhang et al. 2015b), as in former *Calophaca* (Zhang et al. 2015a), *Caragana* (Zhang and Fritsch 2010) and *Hedysarum* (Shue 1985; Duan et al. 2015).

Most previous workers did not accept the generic status of *Chesniella*, treating it within *Chesneya* (Borissova 1945; Li 1981; Rechinger 1984; Zhu and Cao 1986; Fu 1987, 1989; Yakovlev 1988; Yakovlev et al. 1991). Nevertheless, Li (1993) and Zhu and Larsen (2010) stated that the former is distinguishable from the latter by non-reduced stems, membranous stipules, obviously smaller calyxes, flowers and pods. With the inclusion of *Chesneya macrantha* (Fig. 3E), our results justified the monophyly of *Chesniella* (Figs 1, 2), consistent with Zhang et al. (2015b). Within *Chesniella*, two well-supported groups were resolved in our ITS tree (Fig. 1). *Chesniella macrantha* and *Chesneya mongolica* (Maxim.) Boriss. constituted group A, the group B included *Chesniella ferganensis*, *Chesneya gracilis* Boriss. and *Chesneya tribuloides* (Nevski.) Boriss. The former confined in Mongolia and Inner Mongolia of China, to the contrast, the latter ranged from northwestern China to central Asia, which implied vicariance caused by Altai Mountain may drive the divergence of these two groups. However, due to undersampling and distinct morphology of *Chesneya macrantha* in *Chesniella*, the evolution history and infrageneric taxonomy of this genus needs to be further explored.

Yakovlev and Sviazeva (1987) erected *Spongiocarpella* as a segregate genus from *Chesneya* in the light of the former’s spongiose legumes. Such treatment was followed by Yakovlev (1988), Fu (1989) and Yakovlev et al. (1996), but was rejected by Li (1993), Zhu (1996), Qian (1998) and Zhu and Larsen (2010). Based on field and herbarium studies, we concur with Zhu (1996) that the sponge-like pericarp is an unstable character. Additionally, several species formerly assigned to *Spongiocarpella* were represented in our study, including *Chesneya nubigena* (D.Don) Ali, *Chesneya Spinosa* and *Chesniella macrantha*. They did not form a monophyletic group (Figs 1, 2). Thus, our data do not support the generic status of *Spongiocarpella* (as in Zhu 1996; Zhu and Larsen 2010; Ranjbar et al. 2014; Zhang et al. 2015b).

### Monophyly of *Gueldenstaedtia* and *Tibetia*

*Gueldenstaedtia* is a small genus comprised of ca. 10 species and is distinguished from *Chesneya* by its palmately nerved wing petals (vs. pinnately in *Chesneya*) and non-twisted pod valves (vs. twisted) (see Fig. 3G; Liu et al. 2010a). This genus ranges from the Sino-Himalayan region to Mongolia and Siberia (Lock and Schrire 2005; see Fig. 3A). It was established by Fischer (1823) and revised by Fedtschenko (1927), Jacot (1927) and Kitagawa (1936). Ali (1962) divided it into Gueldenstaedtia subg. Gueldenstaedtia and Gueldenstaedtia subg. Tibetia Ali, but the latter was elevated to the generic rank by Tsui (1979) based on characters of stems, stipules, styles and seeds (see Fig. 3H). The genus *Tibetia* was generally accepted in subsequent revisions (Shue 1992; Yakovlev et al. 1996; Cui 1998; Wu 1999; Zhu 2004, 2005a; Bao and Brach 2010), and it is confined to Tibet and the adjacent regions including southern Gansu, southern Qinghai, western Sichuan and northwestern Yunnan of China, northern India, Nepal and Buhtan (Tsui 1979; Grierson and Long 1987; Lock and Schrire 2005; Zhu 2005a; Bao and Brach 2010).

*Gueldenstaedtia* and *Tibetia* were each supported to be monophyletic, and the two genera together form the GUT clade (Figs 1, 2). It seems valid to retain the generic status of each genus, which is also supported by karyological studies (Nie et al. 2002; Yang 2002; Zhu 2005b): *Gueldenstaedtia* (*x* = 7) vs. *Tibetia* (*x* = 8). Within *Gueldenstaedtia*, three species were sampled (all belonging to Gueldenstaedtia sect. Gueldenstaedtia according to Tsui 1979), but these species were all treated to be *Gueldenstaedtia verna* (Georgi) Boriss. *s.l.* by some workers (Yakovlev 1988; Zhu 2004; Bao and Brach 2010). Further work is needed to test the delimitation of *Gueldenstaedtia verna* *s.l.*

Within *Tibetia*, two accessions of *Tibetia himalaica* (Baker) H.P.Tsui grouped together, which were sister to *Tibetia yadongensis* H.P.Tsui (Figs 1, 2). The tree topology and the morphological characters (e.g., elongate stem and round or retuse leaflets apex) seem to be consistent with treating *Tibetia himalaica* as a distinct species (also see Tsui 1979; Cui 1998; Zhu 2005a; Bao and Brach 2010).

## Taxonomic treatment

### Caragana

Taxon classificationPlantaeFabalesLeguminosae

Fabr., Enum. Ed. 2. 421. 1763, emend. nov. L.Duan, J.Wen & Zhao Y.Chang

Calophaca Fisch. ex DC., Prod. 2: 270. 1825, syn. nov. Type: Calophaca wolgarica Fisch., Prod. 2: 270. 1825.
Halimodendron Fisch. ex DC., Prod. 2: 269. 1825, syn. nov. Type: Halimodendron halodendron (Pall.) Druce, Rep. Bot. Soc. Exch. Club Brit. Isles 4: 626. 1917.

#### Type.

*Caragana arborescens* Lam., Encycl. 1(2): 615. 1785.

#### Description.

Shrubs, subshrubs or rarely small trees. Stipules caducous or persistent. Leaves paripinnate, rarely imparipinnate (Chesneya sect. Calophaca), 4–27-foliolate; leaflet blades with margin entire. Lax raceme or fascicled flowers axillary, or flowers solitary. Calyx tubular or campanulate, base usually oblique, teeth 5. Corolla yellow, purple, pink or white; standard ovate to suborbicular, clawed or reflexed at margin; wings and keel often auriculate. Stamens diadelphous (9+1). Ovary sessile to stipitate, with ovule 1-many; style filiform. Pod inflated, compressed, cylindric or linear, dehiscent or rarely indehiscent (Chesneya sect. Halimodendron), with twisted or thickened valve.

#### Distribution and habitat.

This genus contains ca. 100 species, ranging from eastern Europe, Caucasus, western and central Asia, Sino-Himalayan region to Mongolia and Siberia.

### Caragana sect. Calophaca

Taxon classificationPlantaeFabalesLeguminosae

 (Fisch. ex DC.) L.Duan, J.Wen & Zhao Y.Chang stat. & comb. nov.

urn:lsid:ipni.org:names:77157989-1

Calophaca Fisch. ex DC., Prod. 2: 270. 1825. Type: Calophaca wolgarica Fisch., Prod. 2: 270. 1825.

#### Distribution and habitat.

This section includes 5–8 species, distributed in Caucasus, central Asia, northwestern Xinjaing, Innner Mongolia and Shanxi of China.

### Caragana sect. Halimodendron

Taxon classificationPlantaeFabalesLeguminosae

(Fisch .ex DC.) L.Duan, J.Wen & Zhao Y.Chang stat. & comb. nov.

urn:lsid:ipni.org:names:77157990-1

Halimodendron Fisch. ex DC., Prod. 2: 269. 1825. Type: Halimodendron halodendron (Pall.) Druce, Rep. Bot. Soc. Exch. Club Brit. Isles 4: 626. 1917.

#### Type.

*Caragana halodendron* (Pallas) Dumont de Courset, Bot. Cult. 3: 513. 1802.

#### Distribution and habitat.

This section is monotypic and distributes in Caucasus, northeastern Turkey, northern Iran, northern Afghanistan, northern Pakistan, central Asia, western Mongolia, Shanxi and Xinjiang of China.

### Key to the sections of *Caragana*

**Table:** 

1	Leaves imparipinnate; ovary sessile	**Caragana sect. Calophaca**
–	Leaves paripinnate; ovary subsessile or stipitate	**2**
2	Racemose; pedicel non-articulate; pods inflated, indehiscent, valve thickened; seeds few	**Caragana sect. Halimodendron**
–	2–5 flowers in fascicles, or solitary flower; pedicel articulate; pods compressed, cylindric or linear, dehiscent, valve twisted; seeds many	**3**
3	Petiole and rachis always caducous; leaves pinnate	**Caragana sect. Caragana**
–	Petiole and rachis persistent, usually spinelike; leaves pinnate or digitate	**4**
4	Leave digitate	**Caragana sect. Frutescentes**
–	Leave pinnate or partly digitate	**5**
5	Leave digitate or pinnate with 4 leaflets on short branchlets, leave pinnate on long branchlets	**Caragana sect. Spinosae**
–	Leaves pinnate	**6**
6	Petiole and rachis persistent	**Caragana sect. Jubatae**
–	Petiole and rachis persistent on long branchlets, caducous on short branchlets	**Caragana sect. Bracteolatae**

### Chesneya

Taxon classificationPlantaeFabalesLeguminosae

Lindl. ex Endl., Gen.: 1275. 1840.

Fig. 3B–D

Spongiocarpella Yakovlev & N.Ulziykhutag, Bot. Zhur. 17(2): 249. 1987. syn. nov. Type: Spongiocarpella nubigena (D.Don) Yakovl., Bot. Zhur. 17(2): 249. 1987, based on Chesneya nubigena (D.Don) Ali. (see blow)

#### Type.

*Chesneya rytidosperma* Jaub. et Spach, Ill. Pl. Orient. 1(5): 93. 1842.

### Chesneya sect. Chesneya

Taxon classificationPlantaeFabalesLeguminosae

Fig. 3B

Chesneya sect. Macrocarpon Boriss., Fl. U.S.S.R. 11: 280. 1945. syn. nov. Type: Chesneya rytidosperma Jaub. et Spach, Ill. Pl. Orient. 1(5): 93. 1842.

#### Description, distribution and habitat.

This section includes the majority of *Chesneya* species. It can be diagnosed by reduced stems and caducous petiole and rachis. It contains ca. 20 xeric species, ranging from desert and dry slope of northwestern China and western Tibet to central and western Asia and Caucasus.

### Chesneya sect. Pulvinatae

Taxon classificationPlantaeFabalesLeguminosae

M.L.Zhang, Biochem. Syst. Ecol. 63: 89. 2015.

Fig. 3D

Spongiocarpella Yakovlev & N. Ulziykhutag, Bot. Zhur. 17(2): 249. 1987. Type: Spongiocarpella nubigena (D.Don) Yakovl., Bot. Zhur. 17(2): 249. 1987.

#### Type.

*Chesneya nubigena* (D.Don) Ali, Scientist (Karachi) iii: 4. 1959.

#### Description, distribution and habitat.

This psychric section is composed of *Chesneya nubigena*, *Chesneya polystichoides* (Hand.-Mazz.) Ali and *Chesneya purpurea*. It differs from other sections by blackened, curved and non-spiny petiole and rachis, distributed on high-altitude slope in eastern Himalayas and southern and eastern Tibet.

### Chesneya sect. Spinosae

Taxon classificationPlantaeFabalesLeguminosae

L.Duan, J.Wen & Zhao Y.Chang sect. nov.

urn:lsid:ipni.org:names:77157991-1

Fig. 3C

#### Type.

*Chesneya spinosa* P.C.Li, Acta Phytotax. Sin. 19(2): 236. 1981.

#### Description, distribution and habitat.

This monotypic section is recognized by its hardened-spiny petiole and rachis. It is restricted in high-altitude psychrophytic rocky slope in southern Tibet.

### Key to the sections of *Chesneya*

**Table:** 

1	Plant non-pulvinate, petiole and rachis caducous, leaflet apices truncate or emarginate	**Chesneya sect. Chesneya**
–	Plant pulvinate, petiole and rachis persistent, leaflet apices acute	**2**
2	Petiole and rachis hardened and spiny, leaflet apices with short spines	**Chesneya sect. Spinosae**
–	Petiole and rachis blackened and curved, leaflet apices without short spines	**Chesneya sect. Pulvinatae**

### Chesniella macrantha

Taxon classificationPlantaeFabalesLeguminosae

(Cheng f. ex H.C.Fu) L.Duan, J.Wen & Zhao Y.Chang comb. nov.

urn:lsid:ipni.org:names:77157988-1

Chesneya macrantha Cheng f. ex H.C.Fu, Fl. Intramongol. 3: 291. 1977.

#### Note.

Information of the type specimen was not included in its protolog, which was recorded in Acta Phytotax. Sin. 19(2): 237. 1981: China. Inner Mongolia: Baganmao, 29 May 1931, *T.N.Liou 2146* (holotype: PE!).

#### Specimens examined.

**CHINA. Ningxia**: Mt. Helan, 1200m, May 15 1923, *R.C.Ching 108* (US); **Inner Mongolia**: Alasan Left Banner, Xiazi valley, 24 Apr 2009, *Z.Y.Chang et al. 2009054* (WUK); Mt. Yabulai, Agui temple, 1300m, Apr 26 2008, *L.R.Xu 2008008* (WUK); **Xinjiang**: Qomul, 43° 05.330’N, 93° 42.030’E, 1311m, 6 Jun 2004, *Z.Y.Chang et al. 2004516* (WUK).

#### Distribution and habitat.

Dry slopes in Mongolia and Inner Mongolia, Ningxia and Xinjiang of China.

## Supplementary Material

XML Treatment for Caragana

XML Treatment for Caragana sect. Calophaca

XML Treatment for Caragana sect. Halimodendron

XML Treatment for Chesneya

XML Treatment for Chesneya sect. Chesneya

XML Treatment for Chesneya sect. Pulvinatae

XML Treatment for Chesneya sect. Spinosae

XML Treatment for Chesniella macrantha

## Supplementary materials

Supplementary material 1 Voucher information and GenBank accession numbers Data type: Multi-records Explanation note: Data are arranged in the order: taxon name, locality, collector(s), collection number and herbarium, GenBank accession numbers for ITS, *matK*, *trnL-trnF*, *psbA-trnH*. Newly generated sequences are indicated by an asterisk (*); missing sequences are indicated by a dash (–). This dataset is made available under the Open Database License (http://opendatacommons.org/licenses/odbl/1.0/). The Open Database License (ODbL) is a license agreement intended to allow users to freely share, modify, and use this Dataset while maintaining this same freedom for others, provided that the original source and author(s) are credited.Lei Duan, Xue Yang, Peiliang Liu, Gabriel Johnson, Jun Wen, Zhaoyang Chang
