# Supplementary material for: A molecular phylogeny of Caraganeae (Leguminosae, Papilionoideae) reveals insights into new generic and infrageneric delimitations
Source: PhytoKeys. 2016 Oct 4;(70):111–37. doi: 10.3897/phytokeys.70.9641 (PMC5088706; doi:10.3897/phytokeys.70.9641)
Supplement: Supplementary material 1 — Voucher information and GenBank accession numbers [file phytokeys-070-111-s001.docx]

**Supplementary file 1.** Voucher information and GenBank accession numbers used in this study. Data are in the order: taxon name, locality, collector(s), collection number and herbarium, GenBank accession numbers for ITS, *matK*, *trnL-trnF*, *psbA-trnH*. Newly generated sequences are indicated by an asterisk (*); missing sequences are indicated by a dash (–).

*Alhagi sparsifolia* Shaparenko ex Keller & Shaparenko, China: Xinjiang, *S. M. Duan TLF001* (TURP), -, AY177669^1^, KP338225*, KP338351*. *Astragalus rhizanthus* Royle ex Bentham, China: Tibet, *Z. Y. Chang et al. 2011266* (WUK), KP338104*, KP338482*, KP338226*, KP338352*. *Astragalus strictus* Graham ex Bentham: China, Tibet, *Z. Y. Chang et al. 2011185* (WUK), KP338105*, KP338483*, KP338227*, KP338353*. *Calophaca hovenii* Schrenk, GQ246092^2^, -, -, -. *Calophaca pskemica* Gorbunova, GQ246091^2^, -, -, -. *Calophaca wolgarica* (L.f.) Fisch., GQ246093^2^, -, -, -. *Calophaca sinica* Rehder, China: Shanxi, *Z. Y. Chang et al. 2009157* (WUK), KP338106*, KP338484*, KP338228*, KP338354*. *Calophaca soongorica* Kar. & Kir., FJ537288^3^, -, -, -. *Caragana alpina* Y. X. Liou, China: Tibet, *Y. S. Chen et al. 13-1760* (WUK), KT582467*, KT582434*, KT582418*, KT582450*. *Caragana altaica* (Komarov) Pojarkova, China: Xinjiang, *Z. Y. Chang et al. 2004471* (WUK), KP338107*, KP338485*, KP338229*, KP338355*. *Caragana arborescens* Lamarck, DQ311963^4^, AF142737^5^, GQ890296^6^, GU396766^7^. *Caragana bicolor* Komarov, China: Sichuan, *Z. Y. Chang et al. QZ-211* (WUK), KP338108*, -, -, -. *Caragana brachypoda* Pojarkova, China, Inner Mongolia, *Z. Y. Chang et al. 2009068* (WUK), KT582468*, -, -, -. *Caragana brevifolia* Komarov, China: Gansu, *Z. Y. Chang et al. 2007049* (WUK), KT582469*, KT582435*, KT582419*, KT582451*. *Caragana camilli-schneideri* Komarov, China: Xinjiang, *Z. Y. Chang et al. 2004381* (WUK), KP338109*, KP338487*, KP338230*, KP338357*. *Caragana chinghaiensis* Y. X. Liou, China: Qinghai, *Z. Y. Chang et al. 2010160* (WUK), KP338110*, KP338488*, KP338231*, KP338358*. *Caragana crassispina* C. Marquand, China: Tibet, *Z. Y. Chang et al. QZ-584* (WUK), KP338111*, KP338489*, KP338232*, KP338359*. *Caragana densa* Komarov, China: Sichuan, *Z. Y. Chang et al. QZ-312* (WUK), KT582470*, KT582436*, KT582420*, KT582452*. *Caragana erinacea* Komarov, China: Tibet, *Z. Y. Chang et al. QZ-719* (WUK), KP338112*, KP338490*, KP338233*, KP338360*. *Caragana franchetiana* Komarov, China: Sichuan, *Z. Y. Chang et al. QZ-237* (WUK), KP338113*, KP338491*, KP338234*, KP338361*. *Caragana gerardiana* Bentham, China: Qinghai, *Z. Y. Chang et al. 2010258* (WUK), KP338114*, KP338492*, KP338235*, KP338362*. *Caragana grandiflora* DC., GQ246096^2^, AB854564^8^, AB287412^9^, -. *Caragana* *halodendron* (Pallas) Dumont de Courset, China: Xinjiang, *S. M. Duan TLF002* (TURP), KP338147*, JQ619947^13^, KP338270*, KP338398*. *Caragana kansuensis* Pojarkova, China: Gansu, *Z. Y. Chang et al. 2007032* (WUK), KP338115*, KP338493*, KP338236*, KP338363*. *Caragana kirghisorum* Pojarkova, FJ537280^3^, -, -, -. *Caragana korshinskii* Komarov, China: Inner Mongolia, *Z. Y. Chang et al. 2009080* (WUK), KP338116*, AY189778^10^, KP338237*, KP338364*. *Caragana kozlowii* Komarov, China: Tibet, *Z. Y. Chang et al. QZ-509* (WUK), KP338117*, KP338494*, KP338238*, KP338365*. *Caragana leucophloea* Pojarkova, China: Xinjiang, *Z. Y. Chang et al. 2004138* (WUK), KP338118*, KP338495*, KP338239*, KP338366*. *Caragana leveillei* Komarov, China: Gansu, *Z. Y. Chang et al. 2010001* (WUK), KP338119*, KP338496*, KP338240*, KP338367*. *Caragana licentiana* Handel-Mazzetti, China: Gansu, *Z. Y. Chang et al. 2010002* (WUK), KT582471*, KT582437*, KT582421*, KT582453*. *Caragana opulens* Komarov, China: Shaanxi, *Z. Y. Chang et al. 2009043* (WUK), KP338120*, KP338497*, KP338241*, KP338368*. *Caragana pruinosa* Komarov, China: Xinjiang, *Z. Y. Chang et al. 2004290* (WUK), AB262533^14^, KP338498*, -, KP338369*. *Caragana pumila* Pojarkova, China: Xinjiang, *Z. Y. Chang et al. 2004312* (WUK), KP338121*, KP338499*, KP338242*, KP338370*. *Caragana purdomii* Rehder, China: Shaanxi, *Z. Y. Chang et al. 2010027* (WUK), KP338122*, KP338500*, KP338243*, KP338371*. *Caragana roborovskyi* Komarov, China: Ningxia, *Z. Y. Chang et al. 2009021* (WUK), KP338124*, KP338502*, KP338245*, KP338373*. *Caragana rosea* Turczaninow ex Maximowicz, China: Shanxi, *Z. Y. Chang et al. 2009148* (WUK), KP338125*, HM049542^11^, KP338246*, KP338374*. *Caragana soongorica* Grubov, China: Xinjiang, *Z. Y. Chang et al. 2004260* (WUK), KP338126*, KP338503*, KP338247*, KP338375*. *Caragana spinosa* (Linnaeus) Hornemann, China: Xinjiang, *Z. Y. Chang et al. 2004503* (WUK), KP338127*, KP338504*, KP338248*, KP338376*. *Caragana tangutica* Maximowicz, China: Sichuan, *Z. Y. Chang et al. QZ-459* (WUK), FJ537278^3^, KP338505*, KP338249*, KP338377*. *Caragana tekesiensis* Y. Z. Zhao & D. W. Zhou, China: Xinjiang, *Z. Y. Chang et al. 2004288* (WUK), KT582472*, KT582438*, -, KT582454*. *Caragana tibetica* (Maximowicz ex C. K. Schneider) Komarov, China: Qinghai, *Z. Y. Chang et al. 2010161* (WUK), KP338128*, KP338506*, KP338250*, KP338378*. *Caragana versicolor* Bentham, China: Qinghai, *Z. Y. Chang et al. 2010264* (WUK), KT582473*, KT582439*, KT582422*, KT582455*. *Chesneya acaulis* (Baker) Popov, China: Tibet, *Z. Y. Chang et al. 2013166* (WUK), KT582474*, KT582440*, KT582423*, KT582456*. *Chesneya astragalina* Jaub. & Spach, AB051906^12^, JQ619959^13^, AB287413^9^, JF409800^14^. *Chesneya badachschanica* Boriss., KT834922^15^, -, -, -. *Chesneya borissovae* Pavlov, KT834923^15^, -, -, -. *Chesneya botschantzevii* R.M.Vinogr., KT834924^15^ -, -, -. *C**hesneya crassipes* Boriss., KT834925^15^, -, -, -. *Chesneya cuneata* (Benth.) Ali, GQ246102^2^, -, -, -. *Chesneya darvasica* Boriss., KT834927^15^, -, -, -. *Chesneya dshungarica* Golosk., KT834928^15^, -, -, -. *Chesneya elegans* Fomin, GQ246103^2^, -, -, -. *Chesneya hissarica* Boriss., KT834933^15^, -, -, -. *Chesneya kopetdaghensis* Boriss., KT834934^15^, -, -, -. *Chesneya kotschyi* Boiss., GQ246104^2^, -, -, -. *Chesneya kschtutica* Rassulova & B.A.Sharipova, KT834936^15^, -, -, -. *Chesneya linczevskyi* Boriss., KT834937^15^, -, -, -. *Chesneya macrosperma R. Kam.*, KT834938^15^, -, -, -. *Chesneya neplii* Boriss., KT834940^15^, -, -, -. *Chesneya nubigena* (D. Don) Ali - 1, China: Tibet, *Z. Y. Chang et al. 2011258* (WUK), KT582478*, KT582444*, KT582427*, KT582460*. *Chesneya nubigena* (D. Don) Ali - 2, China: Tibet, *Z. Y. Chang et al. 2013086* (WUK), KT582479*, KT582445*, KT582428*, KT582461*. *Chesneya parviflora* Jaub. & Spach, GQ246101^2^, -, -, -. *Chesneya purpurea* P. C. Li, China: Tibet, *Y. S. Chen et al. 13-1760* (WUK), KT582480*, KT582446*, -, KT582462*. *Chesneya quinata* Fed., KT834943^15^, -, -, -. *Chesneya rytidosperma* Jaub. et Spach, KP230737^13^, -, -, -. *Chesneya spinosa* P. C. Li - 1, China: Tibet, *Z. Y. Chang et al. 2013072* (WUK), KT582481*, KT582447*, KT582429*, KT582463*. *Chesneya spinosa* P. C. Li - 2, China: Tibet, *Z. Y. Chang et al. QZ-699* (WUK), KP338130*, KP338508*, KP338252*, KP338380*. *Chesneya ternate* Popow, KT834947^15^, -, -, -. *Chesneya trijuga* Boriss., KT834950^15^, -, -, -. *Chesneya turkestanica* Franch., KT834952^15^, -, -, -. *Chesniella ferganensis* (Korsh.) Boriss., China: Gansu, *Z. Y. Chang et al. 2010379* (WUK), KT582475*, KT582441*, KT582424*, KT582457*. *Chesniella gracilis* Boriss., KT834932^15^, -, -, -. *Chesniella macrantha* (Cheng f. ex H.C.Fu) L.Duan, J.Wen & Zhao Y.Chang - 1, China: Xinjiang, *Z. Y. Chang et al. 2004516* (WUK), KT582476*, KT582442*, KT582425*, KT582458*. *Chesniella macrantha* (Cheng f. ex H.C.Fu) L.Duan, J.Wen & Zhao Y.Chang - 2, China: Inner Mongolia, *Z. Y. Chang et al. 2009054* (WUK), KT582477*, KT582443*, KT582426*, KT582459*. *Chesniella mongolica* (Maxim.) Boriss., KT834939^15^, -, -, -. *Chesniella tribuloides* (Nevski.) Boriss., KT834948^15^, -, -, -. *Cicer microphyllum* Royle ex Bentham, China: Shaanxi, *Z. Y. Chang et al. 2011267* (WUK), KP338131*, KP338509*, KP338253*, KP338381*. *Colutea nepalensis* Sims, China: Tibet, *Z. Y. Chang et al. 2013170* (WUK), KP338132*, KP338510*, KP338254*, KP338382*. *Dalbergia hupeana* Hance, China: Shaanxi, *L. Duan 2012206* (WUK), KP338139*, KP338517*, KP338261*, KP338389*. *Eremosparton songoricum* (Litvinov) Vassilczenko, China: Xinjiang, *WXY tgs705* (XJBI), KP338143*, KP338521*, KP338265*, KP338393*. *Galega officinalis* L., DQ311965^4^, JQ669610^13^, DQ311693^4^, -. *Galega orientalis* Lam., DQ311966^4^, AF522083^16^, DQ311694^4^, -. *Glycyrrhiza lepidota* Pursh, USA: Montana, *Hess et al. 9195* (F), KP338145*, KP338523*, KP338267*, KP338395*. *Glycyrrhiza uralensis* Fischer ex Candolle, China: Shaanxi, *L. Duan 2012205* (WUK), KP338146*, KP338524*, KP338268*, KP338396*. *Gueldenstaedtia multiflora* Bunge., China, Inner Mongolia, *Z. Y. Chang et al. 2003237* (WUK), KT582432*, KP338525*, KP338269*, KP338397*. *Gueldenstaedtia stenophylla* Bunge., GQ246109^2^, JQ669620^13^, AB854532^8^, GU396774^7^. *Gueldenstaedtia verna* (Georgi) Borissova, GQ246106^2^, JQ619946^13^, AB854533^8^, -. *Hedysarum chinense* (B. Fetsch.) Hand. -Mazz., China: Shaanxi, *Z. M. Jiang 1565* (WUK), KP338159*, KP338536*, KP338280*, KP338412*. *Hedysarum petrovii* Yakovlev, China: Ningxia, *Z. Y. Chang et al. 2012102* (WUK), KP338181*, KP338557*, KP338299*, KP338433*. *Lathyrus latifolius* L., China: Shaanxi, *L. Duan 2012196* (WUK), KP338193*, KP338569*, KP338311*, KP338445*. *Lessertia frutescens* (L.) Goldblatt & J.C.Manning, Mexico: Puebla, *P. Tenorio* *L. 18282* (F), KP338216*, KP338596*, KP338341*, KP338473*. *Onobrychis arenaria* DC., Greece: Ipiros, *P. Newman et al. 31/2* (K), KP338194*, KP338570*, KP338312*, KP338446*. *Oxytropis microphylla* (Pallas) Candolle, China: Tibet, *Z. Y. Chang et al. 2011294* (WUK), KP338205*, KP338584*, KP338328*, KP338460*. *Robinia pseudoacacia* L., China: Shaanxi, *L. Duan 2012193* (WUK), KP338206*, AF142728^5^, KP338329*, KP338461*. *Sulla capitata* (Desf.) B. H. Choi & H. Ohash, Tunisia, *C. J. Pitard 114* (US), KP338214*, KP338592*, KP338337*, KP338469*. *Swainsona salsula* (Pall.) Taubert, USA: Oregon, *R. Spellenberg 327* (F), KP338217*, KP338597*, KP338342*, KP338474*. *Tibetia himalaica* (Baker) H. P. Tsui - 1, China: Tibet, *Z. Y. Chang et al. 2011085* (WUK), KT582482*, -, KT582430*, KT582464*. *Tibetia himalaica* (Baker) H. P. Tsui - 2, China: Tibet, *Z. Y. Chang et al. 2011138*(WUK), KT582483*, -, KT582431*, KT582465*. *Tibetia yadongensis* H. P. Tsui, China: Tibet, *Z. Y. Chang et al. 2011142* (WUK), KP338222*, KT582448*, KP338348*, KP338479*. *Trifolium repens* L., China: Shaanxi, *L. Duan 2012195* (WUK), KP338223*, KP338602*, KP338349*, KP338480*. *Wisteria sinensis* (Sims) Sweet, China: Shaanxi, *L. Duan 2012194* (WUK), KP338224*, KP338603*, KP338350*, KP338481*.

Note: Sequences obtained from other studies are listed as follows. ^1^W. B. Yin *et al.* unpublished; ^2^T. K. Ahlquist & M. F. Wojciechowski, unpublished; ^3^M. L. Zhang, P. W. Fritsch & B. C. Cruz, 2009; ^4^N. W. Ellison *et al.* 2006; ^5^J. M. Hu *et al.* 2000; ^6^M. Zhang, Y. Kang & J. Yang, unpublished; ^7^S. L. Chen & T. Gao, unpublished; ^8^A. Amirahmadi *et al.* 2014; ^9^S. K. Osaloo et al. 2006; ^10^J. Hu *et al.* direct submission; ^11^T. Gao *et al.* 2011; ^12^S. K. Osaloo *et al.* 2003; ^13^M. F. Wojciechowski, unpublished; ^14^M. Riahi *et al.* 2011; ^15^Zhang et al., 2015b; ^16^K. P. Steele & M. F. Wojciechowski, unpublished.
